# A Neural Crest-specific Overexpression Mouse Model Reveals the Transcriptional Regulatory Effects of Dlx2 During Maxillary Process Development

**DOI:** 10.3389/fphys.2022.855959

**Published:** 2022-04-21

**Authors:** Jian Sun, NaYoung Ha, Zhixu Liu, Qian Bian, Xudong Wang

**Affiliations:** ^1^ Department of Oral and Cranio-Maxillofacial Surgery, Shanghai Ninth People’s Hospital, Shanghai Jiao Tong University School of Medicine, Shanghai, China; ^2^ National Clinical Research Center for Oral Diseases, Shanghai Key Laboratory of Stomatology, Shanghai Research Institute of Stomatology, Shanghai, China; ^3^ Shanghai Institute of Precision Medicine, Shanghai, China

**Keywords:** cranial neural crest cells, Dlx2, cleft palate, RNA-seq, craniofacial development

## Abstract

Craniofacial morphogenesis is a complex process that requires precise regulation of cell proliferation, migration, and differentiation. Perturbations of this process cause a series of craniofacial deformities. Dlx2 is a critical transcription factor that regulates the development of the first branchial arch. However, the transcriptional regulatory functions of Dlx2 during craniofacial development have been poorly understood due to the lack of animal models in which the Dlx2 level can be precisely modulated. In this study, we constructed a Rosa26 site-directed Dlx2 gene knock-in mouse model *Rosa26* ^*CAG-LSL-Dlx2−3xFlag*^ for conditionally overexpressing Dlx2. By breeding with *wnt1* ^*cre*^ mice, we obtained *wnt1* ^*cre*^ *; Rosa26* ^*Dlx2/-*^ mice, in which Dlx2 is overexpressed in neural crest lineage at approximately three times the endogenous level. The *wnt1* ^*cre*^ *; Rosa26* ^*Dlx2/-*^ mice exhibited consistent phenotypes that include cleft palate across generations and individual animals. Using this model, we demonstrated that Dlx2 caused cleft palate by affecting maxillary growth and uplift in the early-stage development of maxillary prominences. By performing bulk RNA-sequencing, we demonstrated that Dlx2 overexpression induced significant changes in many genes associated with critical developmental pathways. In summary, our novel mouse model provides a reliable and consistent system for investigating Dlx2 functions during development and for elucidating the gene regulatory networks underlying craniofacial development.

## Introduction

Craniofacial morphogenesis is a complex process that requires precise regulation of cell growth, migration, and differentiation. Perturbation of this process leads to a large number of craniofacial abnormalities, including cleft lip, cleft palate, and hemifacial microsomia (Walker & Trainor, 2006). Most craniofacial tissues originate from the neural crest, a transient, multipotent cell population that is first observed during human embryogenesis at Carnegie stage 9 (Hackland et al., 2017). The cranial neural crest cells (CNCCs) migrate to different pharyngeal arches and eventually differentiate into a variety of tissue types (Szabo-Rogers, Smithers, Yakob, & Liu, 2010). The induction, migration, and differentiation of CNCCs require the cooperative activities of many signaling pathways and transcription factors (Schwend & Ahlgren, 2009; Everson et al., 2017; Everson, Fink, Chung, Sun, & Lipinski, 2018). Among the critical factors is the Distal-less homeobox (Dlx) gene family, which consists of six members (Dlx1- Dlx6) and are organized into three convergent pairs on the second chromosome of mice (Depew, Simpson, Morasso, & Rubenstein, 2005). Among the Dlx family transcription factors, Dlx2 is a very important candidate gene that regulates the differentiation and development of the first branchial arch.

During craniofacial development, Dlx2 is expressed in the epithelium and the CNCC-derived mesenchyme of the mandibular and maxillary processes (Depew et al., 2005; Jeong et al., 2012). Previous embryology studies have demonstrated that Dlx2 plays a crucial role in maxillary primordium development by regulating the differentiation and patterning of the maxillofacial skeleton. Both the Dlx2-null and overexpressing mice exhibited severe craniofacial malformation (McKeown, Newgreen, & Farlie, 2005; Jeong et al., 2012). Targeted Dlx2 mutation leads to malformations in CNCCs-derived skeletal tissues, which originate in the maxillary region and the proximal second branchial arch. Mice lacking the activities of both Dlx1 and Dlx2 (Dlx1/2^−/−^) exhibit abnormal upper jaw bone formation (Samee, de Vernejoul, & Levi, 2007) and cleft palate (Jeong et al., 2012). Studies have also shown that Dlx2 overexpression can induce neural crest cell (NCC) classification or aggregation (McKeown et al., 2005). Moreover, Dlx2 overexpression *in ovo* disrupts the migration and differentiation of the affected CNCCs and induces the development of ectopic bone components (Gordon, Brinas, Rodda, Bendall, & Farlie, 2010). Despite the extensive documentation of the phenotypes resulting from the gain or loss of Dlx2 function, which genes Dlx2 specifically regulates during craniomaxillofacial development and how the deregulation of these genes leads to developmental defects remain unclear.

Elucidation of the transcriptional regulatory functions of Dlx2 in craniomaxillofacial development requires animal models in which the Dlx2 level can be precisely modulated and in a tissue-specific manner. To this end, a mouse model (Wnt1Cre:iZEG-Dlx2) that overexpresses Dlx2 in NCCs from multi-copy transgenes has been previously constructed. The Wnt1Cre:iZEG-Dlx2 mice displayed decreased CNCCs proliferation and increased apoptosis, abnormal chondrogenesis, and disrupted osteogenesis, and exhibited a variety of clear developmental defects ranging from a cleft lip and midfacial clefts to neural tube defects and exencephaly, nasal and premaxillary hypoplasia, and spinal deformities (Dai et al., 2013; Dai et al., 2016; Dai et al., 2017). These findings suggest that Dlx2 overexpression in NCCs may be a pathological cause of facial fissures and kyphosis in mammals. However, the high copy-number transgenes result in a very high level of ectopic Dlx2 expression that may not reflect the Dlx2 dysregulation within the physiological contexts. Furthermore, the expression from the multi-copy, plasmid-based transgenes tends to be variable and unstable over time, which may be attributed to the instability of the transgenes and epigenetic silencing. In aggreement with this, the Wnt1Cre:iZEG-Dlx2 mice exhibited a less than 50% phenotypic penetrance rate of craniofacial dysplasia and significant phenotype variations (Dai et al., 2013). These developmental abnormalities gradually diminished after maintaining the iZEG-Dlx2 founder mice over multiple generations, thereby preventing the detailed characterization of the downstream transcriptional effects of Dlx2 overexpression. To overcome these limitations, a Dlx2 overexpression mouse model with more stable phenotypic manifestations and inheritance is needed.

In this study, we engineered a Rosa26 site-directed Dlx2 gene knock-in mouse model *Rosa26* ^*CAG-LSL-Dlx2−3xFlag*^ that can be employed to achieve conditional Dlx2 overexpression. By crossing these mice with *wnt1* ^*cre*^ mice, we obtained *wnt1* ^*cre*^ *; Rosa26* ^*Dlx2/-*^ mice in which Dlx2 is stably overexpressed in the neural crest lineages. The *wnt1* ^*cre*^ *; Rosa26* ^*Dlx2/-*^ mice exhibit a stable cleft palate phenotype with 100% penetrance while dispalying minimal phenotypic variations among different mice. Further analyses revealed that the cleft palate phenotype could be readily observed on embryonic day 15.5 (E15.5). Hyaluronic Acid (HA) staining implied the impairment of maxillary process development prior to palate fusion. By performing bulk RNA-seq (RNA-sequencing) on wild-type (WT) and Dlx2-overexpressing E12.5 maxillary process, we demonstrated that Dlx2 overexpression by approximately 3-fold was sufficient to induce significant changes of thousands of genes within the maxillary processes, among which many genes are associated with critical developmental pathways. Taken together, our novel mouse model provides an important resource for elucidating the physiological and pathological functions of Dlx2 and elucidating the gene regulatory networks during craniofacial development.

## Materials and Methods

### Generation of the *Rosa26* ^*CAG-LSL-Dlx2−3xFlag*^ Mouse Model

**FIGURE 1:**
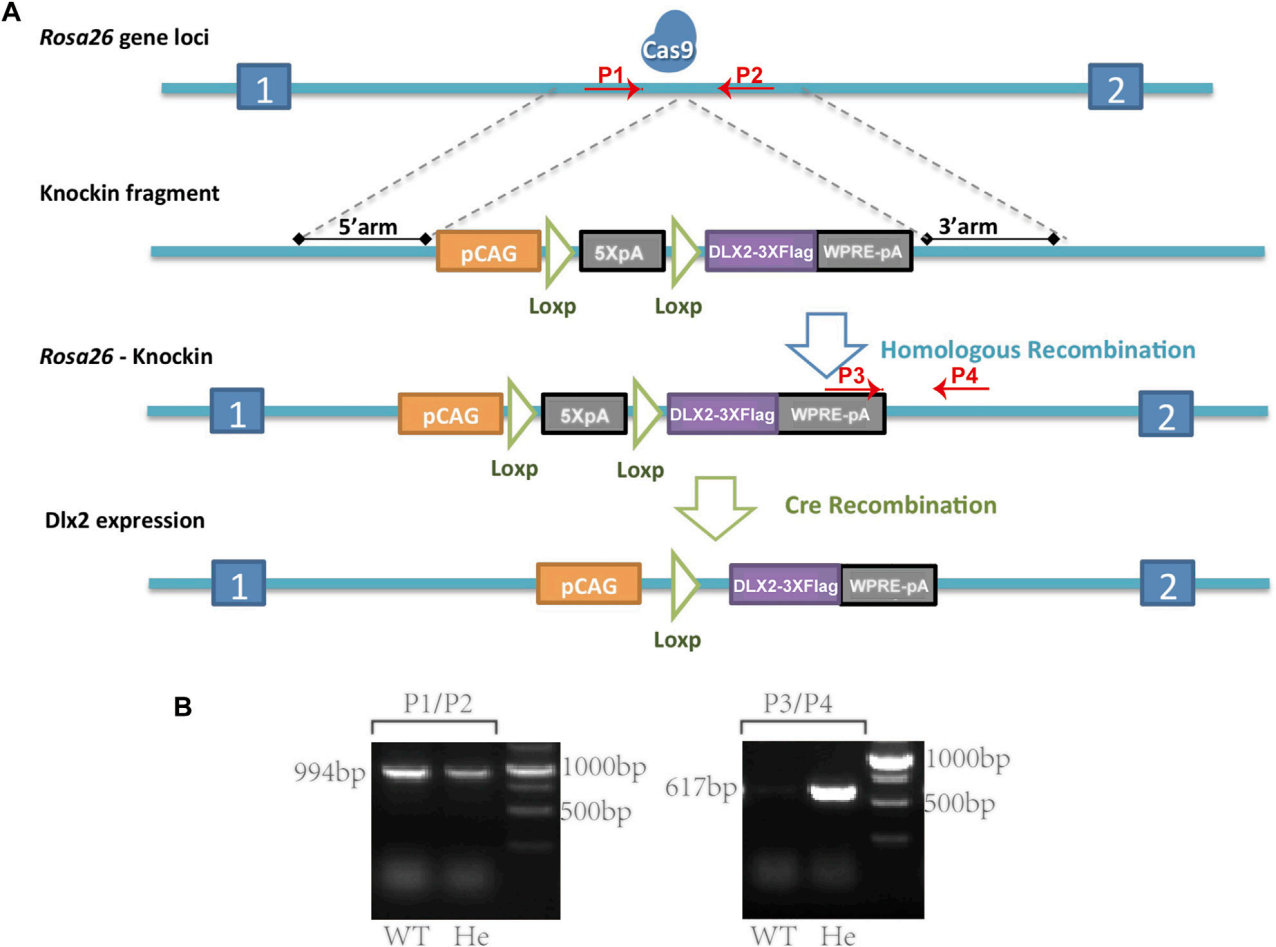
A *Rosa26* ^*CAG-LSL-Dlx2−3xFlag*^ mouse model for conditional Dlx2 overexpression **(A)** Cartoons illustrate the design strategy for conditionally overexpressing Dlx2 by breeding *Rosa26* ^*CAG-LSL-Dlx2−3xFlag*^ mice with Cre mice **(B)** In WT mice (P1, P2) generated a 994 bp band, whereas (P3, P4) did not generate a PCR product. In heterozygous mice (P1, P2) amplified a 994 bp PCR product, while (P3, P4) amplified a smaller PCR product of 617 bp. Cas9, CRISPR/Cas9 system; pCAG, CAG promoter; 5XpA, 5X PolyA (containing STOP cassette); DLX2-3XFlag, coding sequence of Dlx2 with 3X flag; WPRE-pA, woodchuck hepatitis virus post-transcriptional regulatory element (WPRE)-bGH polyA; WT, wild-type; He, heterozygote.

The *Rosa26* ^*CAG-LSL-Dlx2−3xFlag*^ mouse model was developed by Shanghai Model Organisms Center, Inc(Shanghai, China). This model was generated using the CRISPR/Cas9 system in a C57BL/6J mouse background (Figure 1A). The sequence information used in the construction of the mouse model is NM_010,054.2→NP_034,184.1 homeobox protein DLX-2. The knockin site was Gt (ROSA)26Sor (MGI number: 104,735). The chromosome position of the Rosa26 gene is in Chromosome 6: 113,067,428–113,077,333 reverse strand.

Briefly, a targeting vector containing the following components was constructed: CAG-LSL-Dlx2-3xFlag-woodchuck hepatitis virus post-transcriptional regulatory element (WPRE)-bGH poly(A). Cas9 mRNA was *in vitro* transcribed with the mMESSAGE mMACHINET7 Ultra Kit (Ambion, TX, United States) according to the manufacturer’s instructions, and subsequently purified using the MEGAclear™ Kit (ThermoFisher, United States of America). 5′-GGG​GAC​ACA​CTA​AGG​GAG​CT-3′ was chosen as Cas9 targeted guide RNA (sgRNA) and *in vitro* transcribed using the MEGAshortscript Kit (ThermoFisher, United States) and subsequently purified using MEGAclear™ Kit. The donor vector with sgRNA and Cas9 mRNA was microinjected into C57BL/6J fertilized eggs. F0 generation mice positive for homologous recombination were identified by long PCR. The primers (P1-P4) used for genotyping the correct homology recombination were P1: 5′-GCC​GGG​CCT​CGT​CGT​CT-3′ and P2: 5′-TTT​TTG​GGG​GTG​ATG​GTG​GTC-3′ for the correct 5′ homology arm recombination, and P3: 5′-CTG​CGC​GGG​ACG​TCC​TTC​TGC​TAC-3′ and P4: 5′-GGG​ATC​CAT​TGC​CAC​CTT​TCA​CTT-3′ for the correct 3′ homology arm recombination. The PCR products were further confirmed by sequencing. F0 mice were crossed with C57BL/6J mice to obtain *Rosa26* ^*CAG-LSL-Dlx2−3xFlag*^ heterozygous mice.

After the *Rosa26* ^*CAG-LSL-Dlx2−3xFlag*^ mouse model was generated, the primer sequences used for genotype identification were P1: 5′-TCA​GAT​TCT​TTT​ATA​GGG​GAC​ACA-3′, P2: 5′-TAA​AGG​CCA​CTC​AAT​GCT​CAC​TAA-3′, P3: 5′-GGT​GTT​GTC​GGG​GAA​ATC​ATC​GTC-3′ and P4: 5′-AGG​AGC​CTG​CCA​AGT​AAC-3’. The primer pairs (P1, P2) and (P3, P4) were amplified separately. In WT mice, only (P1, P2) amplified a PCR product of 994bp, whereas (P3, P4) should not give rise to any PCR product. In heterozygous mice, (P1, P2) amplified a PCR product of 994bp, while (P3, P4) amplified a smaller PCR product of 617bp (Figure 1B).

### Wild-Type Mouse

WT C57BL/6J mice were purchased from Shanghai Jihui Laboratory Animal Care Co.Ltd.(Shanghai, China). All mice were maintained under SPF conditions at the Animal Center of the Ninth People’s Hospital affiliated to Shanghai Jiao Tong University School of Medicine. All animal experiments were performed by following protocols approved by the Animal Care and Usage Committee of the Ninth People’s Hospital affiliated to Shanghai Jiao Tong University School of medicine.

### Skeletal Staining

After removing the skin, internal organs, and back fat on the day of birth, the mice were treated with 95% ethanol overnight and stained with Alcian Blue at 37°C for 42 h. Following shaking and washing twice with 95% ethanol for 1 h and 2% KOH digestion for 2.5 h, the mice were stained with Alizarin Red for 1 h, digested with 1% KOH for 1 day, soaked in gradient glycerol until there were no bubbles, and photographed.

### Histology and Immunofluorescence staining

Entire heads of E15.5 and E13.5 mice embryos were surgically dissected. Following fixation in 4% paraformaldehyde and gradient dehydration in ethanol, the tissues were embedded in paraffin and sections at a thickness of 8 µm were cut and stained with Hematoxylin and Eosin (HE). Immunofluorescence staining was performed with anti-Dlx2 polyclonal antibody (ab272902, Abcam, United States, 1:250), anti-DDDDK tag antibody (ab205606, Abcam, United States, 1:200), goat secondary antibody to rabbit IgG (ab150077, Abcam, 1:500) following a previously described protocol (Ha, Sun, Bian, Wu, & Wang, 2022). Images were captured using an Olympus IX83 inverted microscope.

### HA Staining

The head sections of E13.5 WT and *wnt1* ^*cre*^ *; Rosa26* ^*Dlx2/-*^ mice were dewaxed to water and a HA staining kit (g3710, Solarbio, China) was used for HA staining. Following hyaluronidase treatment for 3 h, the treated sections were soaked with Alcian Blue for 30 min, counterstained with nuclear solid red for 30min, dehydrated and transparent, air dried, and sealed with neutral gum.

### *In situ* Hybridization

*In situ* hybridization was performed on head tissue sections of mouse embryos to detect the Dlx2 transcript levels using the RNAscope^®^ 2.5HD Reagent Kit- RED (322,350, ACDbio, California, US) and the RNAscope^®^ Probe-Mm-DLX2 (555,951, ACDbio, California, US) according to the manufactor’s instructions. Briefly, the slides were incubated twice at room temperature in xylene and then also 100% ethanol, incubated with the RNAscope^®^ hydrogen peroxide, and washed with distilled water. The slides were then transferred to 99°C RNAscope^®^ 1× Target Retrieval Reagent for 15 min. Following rinsing and ethanol, the slides were incubated in RNAscope^®^ protease Plus at 40°C for 30 min. The RNAscope^®^ Probe-Mm-DLX2 probe was applied and the slides were incubated at 40°Cfor 2 h. The slides were incubated in six amplifiers reagents in turn at toom temperature and washed with 1× wash buffer during each interval. Each section was incubated with sufficient RED working solution prepared using a 1:60 ratio of RNAScope Fast RED-B to RNAScope Fast RED-A. The slides were stained with hematoxylin and mounted.

### Bulk RNA-Seq and Data Analysis

Total RNA was extracted using Trizol from freshly dissected E12.5 maxillary prominence tissues from six littermates. For each genotype (WT and *wnt1* ^*cre*^ *; Rosa26* ^*Dlx2/-*^), three independent RNA samples were prepared. RNA purity was tested using the kaiaoK5500^®^ Spectrophotometer (Kaiao, Beijing, China). RNA integrity and concentration were assessed using the RNA Nano 6000 Assay Kit of the Bioanalyzer 2100 system (Agilent Technologies, CA, United States of America). For each sample, 2 μg total RNA was used as input material for the library preparations. Sequencing libraries were generated using the NEBNext^®^ UltraTM RNA Library Prep Kit for Illumina^®^ (#E7530L, NEB, United States of America) following the manufacturer’s recommendations and index codes were added to attribute sequences to each sample. The ligated products were retrieved and PCR amplification was performed to generate sequencing libraries. The RNA concentration of the library was determined using the Qubit^®^ RNA Assay Kit in Qubit^®^ 3.0 to preliminary quantify and then dilute to 1 ng/μL. The insert size was assessed using the Agilent Bioanalyzer 2100 system (Agilent Technologies), and the qualified insert size was accurately quantified using a StepOnePlus™ Real-Time polymerase Chain Reaction (PCR) System. The clustering of the index-coded samples was performed on a cBot cluster generation system using HiSeq PE Cluster Kit v4-cBot-HS (Illumina) according to the manufacturer’s instructions. Following cluster generation, the libraries were sequenced on an Illumina HiSeq X ten platform and 150 bp paired-end reads were generated. The raw reads of RNA-seq libraries were filtered by removing adaptor sequences, contamination, and low-quality (Phred quality <20) reads. Reads quality was assessed using FastQC. Sequenced reads were mapped to the mm10 genome using STAR aligner version 2.7.3a. Reads were counted using htseq-count version 0.12.4 at the union mode. Comparison between the RNA-seq datasets was performed using the DESeq2 package in R. Differentially expressed genes were defined as genes with a Benjamini–Hochberg adjusted *p*-value (padj) of <0.05 and log2 expression fold change (log2FC) > 1 or < −1. Enrichment analysis and visualization of functional profiles (GO and KEGG) of differentially expressed genes were performed using the clusterProfiler package in R.

### Quantitative Real-Time PCR

Total RNA was extracted using the RNeasy Mini Kit (Qiagen, Valencia, CA, United States of America) from freshly dissected E12.5 maxillary prominence tissues from six littermates, following the manufacturer’s instructions. For each genotype (WT and *wnt1* ^*cre*^ *; Rosa26* ^*Dlx2/-*^), three independent RNA samples different from the Bulk RNA-seq were prepared. The reverse transcriptase reaction was performed using the Hifair III first Strand cDNA Synthesis SuperMix for qPCR (11137ES60, Yeasen, China). Fluorescence-based real-time PCR was performed with Hieff qPCR SYBR Green Master Mix (11201ES08, Yeasen, China). The primer sequences were derived from gene sequences available through the PrimerBank or NCBI (Table 1). The relative expression was calculated for each gene by the 2−∆∆Ct method, normalized against β-actin expression, and presented as fold changes relative to the control. All analyses were performed in triplicate. Statistical analysis was performed using an appropriate Student’s t-test when two values were being compared, with *p* < 0.05 considered as significant.

**TABLE 1:** List of primer sets used for RT-qPCR.

Genes	Forward	Reverse
β-actin	CAT​TGC​TGA​CAG​GAT​GCA​GAA​GG	TGC​TGG​AAG​GTG​GAC​AGT​GAG​G
Dlx2	AAC​CAC​GCA​CCA​TCT​ACT​CC	CGC​TTT​TCC​ACA​TCT​TCT​TGA
Msx2	ATC​CCA​GCT​TCT​AGC​CTT​GGA	GAC​AGG​TAC​TGT​TTC​TGG​CGG
Axin2	AAC​CTA​TGC​CCG​TTT​CCT​CT	CTG​GTC​ACC​CAA​CAA​GGA​GT
Ctnna2	ACAAAGGTCCGTCTGGTA	TCC​TTA​GCG​ATC​TGC​TCA​C
Hoxd1	AGT​CCC​ATC​AAA​TCT​GGC​CG	TTC​AAA​GGT​GGG​GAG​CAG​TC
Wnt3a	GGT​CTA​CTA​CGA​GGC​CTC​AC	CAT​CTA​TGC​CAT​GCG​AGC​TC

## Results

### A *Rosa26* ^*CAG-LSL-Dlx2−3xFlag*^ Mouse Model for Conditional Dlx2 Overexpression

Conditionally overexpressing Dlx2 in NCCs using a high copy-number transgenic mouse model (*Wnt1* ^*Cre*^:iZEG-Dlx2) led to a wide range of craniofacial abnormalities, from cleft lip to neural tube defects and exencephaly. These developmental defects with different severity may be attributed to the variable, and possibly leaky Dlx2 expression from the high copy-number transgenes. To avoid this technical problem and more explicitly address the effect of Dlx2 overexpression on craniofacial development, we constructed a new transgenic mouse model in which a CAG-LSL-Dlx2 expression cassette was inserted into the endogenous Rosa26 locus (*Rosa26* ^*CAG-LSL-Dlx2−3xFlag*^) (Figure 1). The *Rosa26* ^*CAG-LSL-Dlx2−3xFlag*^ mice were bred with *wnt1* ^*cre*^ mice to obtain *wnt1* ^*cre*^ *; Rosa26* ^*Dlx2/-*^ mice, which specifically overexpressed Dlx2 in neural crest-derived cells.

### Temporal Dlx2 Expression Pattern Confirms Its Overexpression in the Early-Stage Development of Maxillary Prominences

**FIGURE 2:**
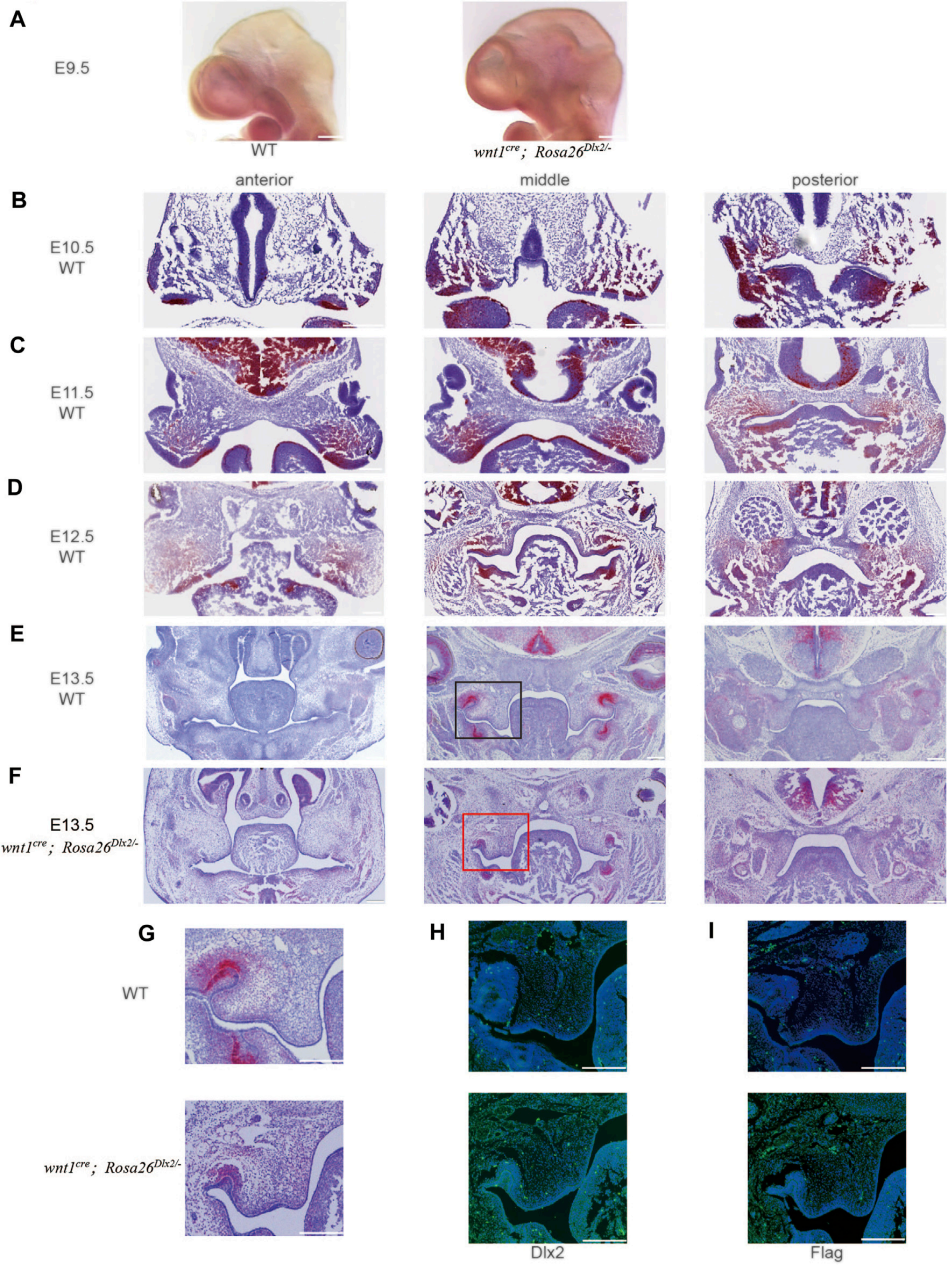
The temporal Dlx2 expression pattern during the early-stage development of maxillary processes from E9.5 to E13.5 **(A)** The whole *in situ* hybridization of E9.5 WT and *wnt1* ^*cre*^ *; Rosa26* ^*Dlx2/-*^ mouse head **(B-E)** *In situ* hybridization on the head tissue sections of E10.5 **(B)**, E11.5 **(C)**, E12.5 **(D)**, and E13.5 **(E)** WT embryos and the temporal Dlx2 expression pattern. Note that Dlx2 expression is largely diminished at E13.5 **(F)** *In situ* hybridization on the head tissue sections of E13.5 *wnt1* ^*cre*^ *; Rosa26* ^*Dlx2/-*^ mouse embryo depicts Dlx2 overexpression in maxillary processes **(G)** Enlarged picture of the box area in **E** and **F (H)** Dlx2 immunofluorescence staining of E13.5 WT (up) and *wnt1* ^*cre*^ *; Rosa26* ^*Dlx2/-*^ (down) mouse maxillary processes **(I)** Flag immunofluorescence staining of E13.5 WT (up) and *wnt1* ^*cre*^ *; Rosa26* ^*Dlx2/-*^ (down) mouse maxillary processes. Bar, 200um.

To further clarify the Dlx2 overexpression, we examined the temporal Dlx2 expression pattern during the early-stage development of maxillary processes from E9.5 to E13.5 (Figure 2).

In the whole *in situ* hybridization of E9.5 mouse head, it can be seen that the expression amount and expression range of Dlx2 in *wnt1* ^*cre*^ *; Rosa26* ^*Dlx2/-*^ mice were increased (Figure 2A). *In situ* hybridization on head tissue sections of E10.5, E11.5, E12.5, and E13.5 WT mouse embryos revealed that Dlx2 was expressed in the epithelium and mesenchyme of the upper and lower jaws of the first branchial arch. At E10.5, the Dlx2 expression was readily detected in the distal region of both the maxilla and mandible of the first branchial arch (Figure 2B). At E11.5, concurrent with the rapid growth of the maxillary prominence, the Dlx2 expression level exhibited a significant elevation compared to that at E10.5. At the same time, the region exhibiting Dlx2 expression extended to the proximal end of the maxilla, occupying a large fraction of the maxillary prominences (Figure 2C). Interestingly, at E12.5, the Dlx2 expression level and area both exhibited a marked reduction (Figure 2D). Dlx2 expression was largely diminished at E13.5 (Figure 2E). Together, these observations revealed that Dlx2 expression in cranial neural crest-derived mesenchyme is largely restricted to E10.5 and E11.5, suggesting that Dlx2 may play a role in promoting the early-stage development of maxillary prominences. These findings also suggest that the ectopic Dlx2 overexpression in *wnt1* ^*cre*^ *; Rosa26* ^*Dlx2/-*^ mice may influence the developmental trajectory of the palate from E12.5 on, when endogenous Dlx2 has been largely downregulated. Compared with E13.5 WT mice, the Dlx2 transcript level in E13.5 *wnt1* ^*cre*^ *; Rosa26* ^*Dlx2/-*^ mice was increased (Figure 2FG). We also performed immunofluorescence staining of Dlx2 and Flag in the maxillary process of E13.5 mice (Figure 2H,I). Dlx2 was also overexpressed at the protein level, which was consistent with the results of *in situ* hybridization.

### NCC-specific Dlx2 Overexpression Using the Rosa26 Locus Knock-In Mouse Model Leads to a Stable Cleft Palate Phenotype

**FIGURE 3:**
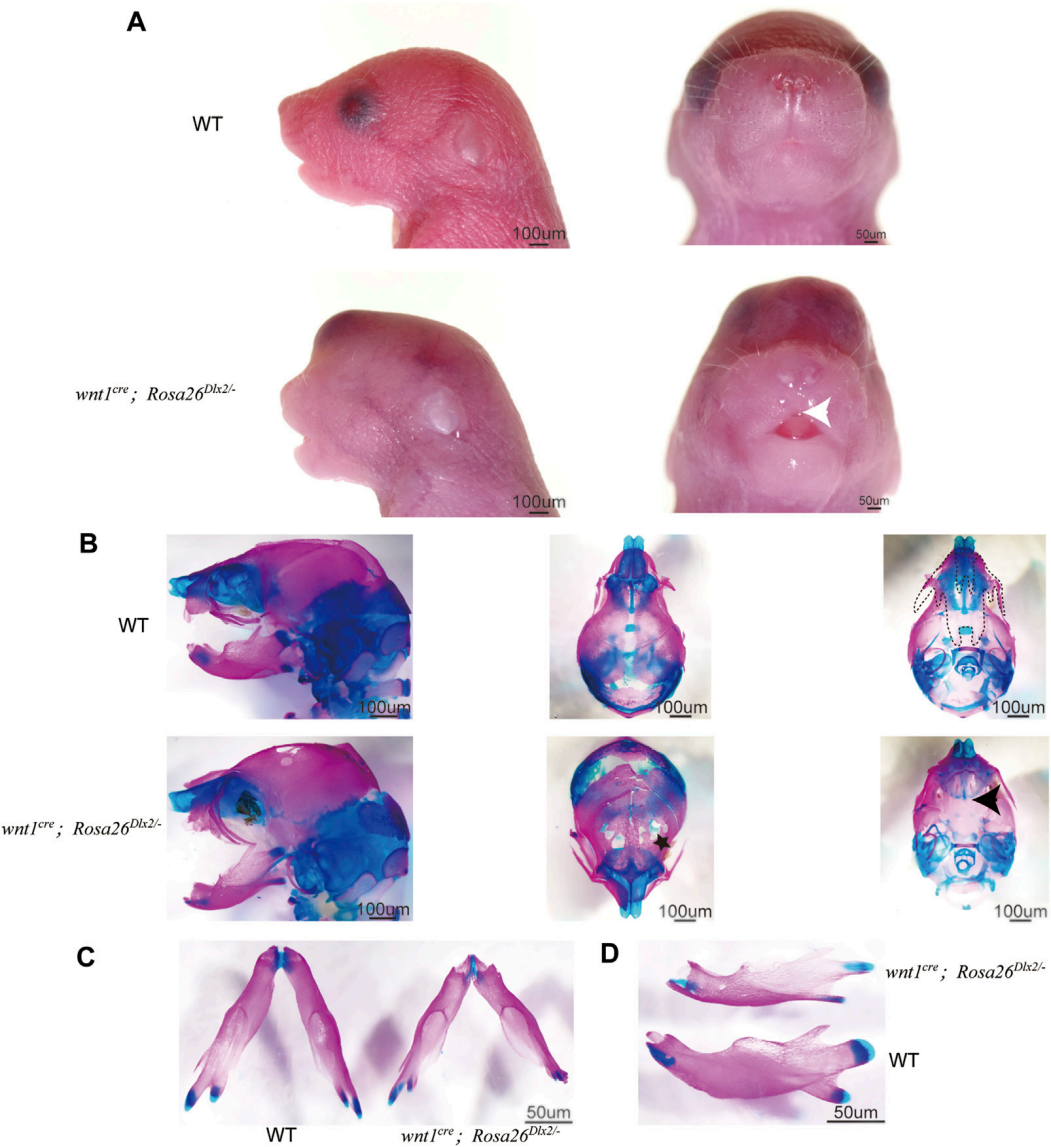
Phenotype of *wnt1* ^*cre*^ *; Rosa26* ^*Dlx2/-*^ mice at P0 **(A)** Stereo microscope images of P0 WT and *wnt1* ^*cre*^ *; Rosa26* ^*Dlx2/-*^ mice. *Wnt1* ^*cre*^ *; Rosa26* ^*Dlx2/-*^ mice exhibit a markedly shorter maxilla, a tiny cleft in the upper lip, abnormal eye development, and soft tissue bulges on the head **(B-D)** Skeletal staining of P0 WT and *wnt1* ^*cre*^ *; Rosa26* ^*Dlx2/-*^ mice shows a range of evident craniofacial abnormality in *wnt1* ^*cre*^ *; Rosa26* ^*Dlx2/-*^ mice **(B)** *Wnt1* ^*cre*^ *; Rosa26* ^*Dlx2/-*^ mice exhibited a partial defect of the skull and parietal bones in addition to the short maxilla and cleft palate **(C)** *Wnt1* ^*cre*^ *; Rosa26* ^*Dlx2/-*^ mice exhibit a shortened mandible **(D)** The temporomandibular joints of the *wnt1* ^*cre*^ *; Rosa26* ^*Dlx2/-*^ mice are abnormally developed. The joint heads are smaller and the articular cartilage is insufficient. The dotted line shows the range of maxilla and palatine, excluding the premaxilla. Black arrows indicate cleft palate, the white arrow points to the tiny cleft in the upper lip, and the black star indicates the partial defect of the frontal bones.

We carefully examined the craniomaxillofacial abnormalities caused by Dlx2 overexpression using this new Rosa26 knock-in model. On the day of birth (P0), *wnt1* ^*cre*^ *; Rosa26* ^*Dlx2/-*^ mice exhibited markedly shorter maxilla compared to their WT littermates, rendering the lips unable to close (Figure 3A). *Wnt1* ^*cre*^ *; Rosa26* ^*Dlx2/-*^ mice also displayed cleft palate (Figure 3B, black arrow), which is consistent with the previously reported phenotypes of *Wnt1* ^*Cre*^:iZEG-Dlx2 mice. Interestingly, the midfacial cleft phenotype associated with the Wnt1Cre:iZEG-Dlx2 mice was not observed in any P0 *wnt1* ^*cre*^ *; Rosa26* ^*Dlx2/-*^ mice. Instead, the *wnt1* ^*cre*^ *; Rosa26* ^*Dlx2/-*^ mice consistently exhibited a tiny cleft in the upper lip (Figure 3A, white arrow), considering the phenotype of previous mouse models, the manifestation of the midfacial cleft may dependent on the dose of the Dlx2 expression.

In addition to the short maxilla and cleft palate, the *wnt1* ^*cre*^ *; Rosa26* ^*Dlx2/-*^ mice displayed abnormal eye development and soft tissue bulges on the head (Figure 3A). Skeletal staining of P0 *wnt1* ^*cre*^ *; Rosa26* ^*Dlx2/-*^ mice revealed a partial defect of the frontal bones (Figure 3B, black star), suggesting that the swelling of the head tissues and the bulging out of the brain could be attributed to defects in skeletal development. The mandible of *wnt1* ^*cre*^ *; Rosa26* ^*Dlx2/-*^ mice was also shortened to some extent (Figure 3C,D). Moreover, the temporomandibular joints of the *wnt1* ^*cre*^ *; Rosa26* ^*Dlx2/-*^ mice were abnormally developed, with smaller joint heads and reduced articular cartilage (Figure 3D). These observations on P0 *wnt1* ^*cre*^ *; Rosa26* ^*Dlx2/-*^ mice are correspond with the previous reports that the three-month-old *Wnt1* ^*Cre*^:iZEG-Dlx2 mice exhibited postnatal condyle malformation, subchondral bone degradation, and an irregular histological structure of the condylar cartilage (Dai et al., 2016), suggesting that Dlx2 plays critical roles in regulating condyle development during embryogenesis. Together, these results revealed that Dlx2 overexpression exerts a profound effect on a diverse array of neural crest-derived craniofacial skeletal tissues.

Of particular note, we found that all six P0 *wnt1* ^*cre*^ *; Rosa26* ^*Dlx2/-*^ mice from three litters of newborn mice (22 in total) across three generations exhibited highly consistent craniofacial abnormalities, including short maxilla and cleft palate. Such a nearly homogeneous phenotypic manifestation demonstrates the stability of our mouse model. The *wnt1* ^*cre*^ *; Rosa26* ^*Dlx2/-*^ mouse model enables further elucidation of Dlx2 function in the regulation of craniofacial development with greater spatiotemporal precision.

### Conditional Dlx2 Overexpression in *wnt1* ^*cre*^ *; Rosa26* ^*Dlx2/-*^ Mice Caused Cleft Palate by Affecting Maxillary Growth and Uplift

**FIGURE 4:**
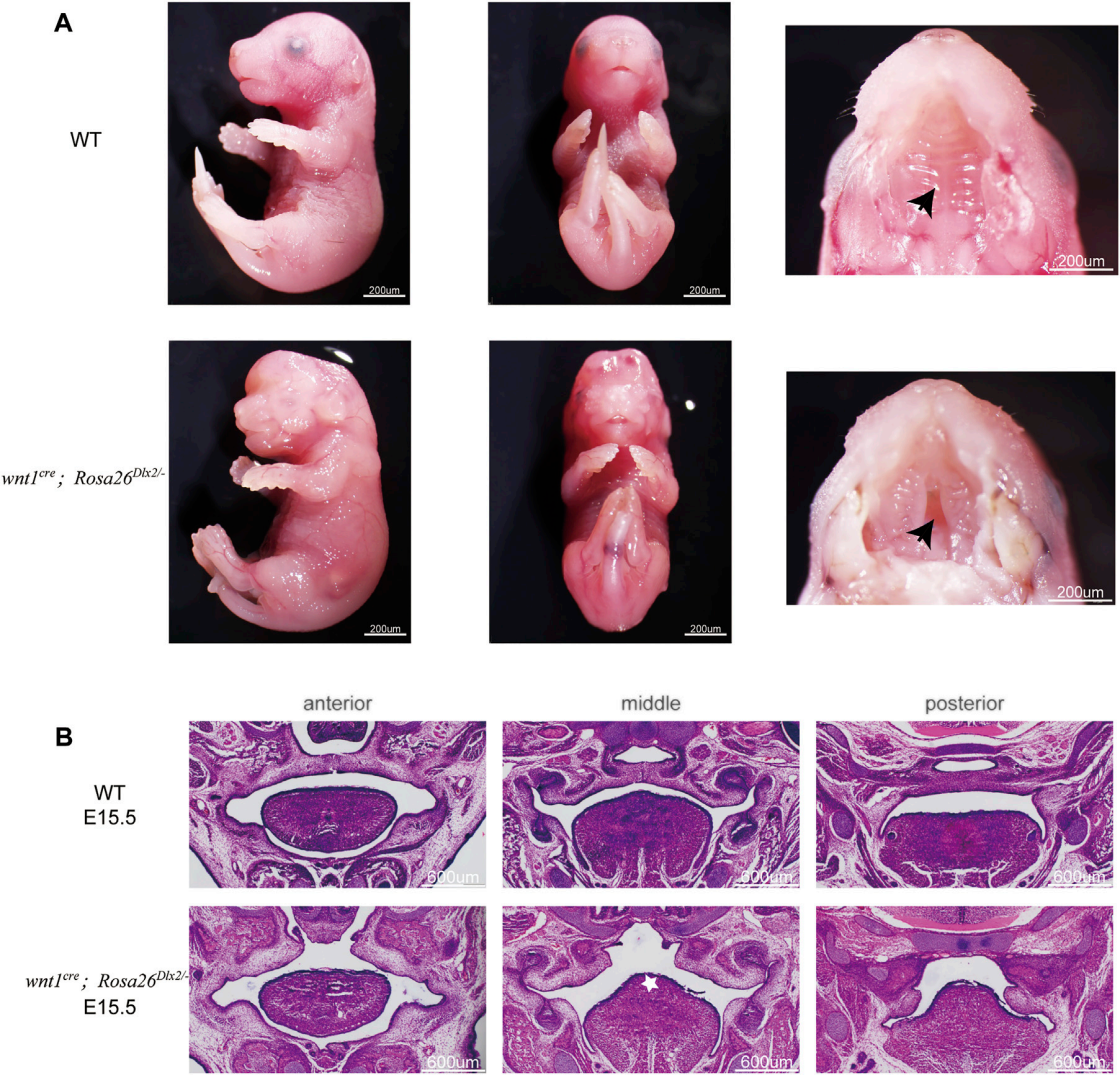
Phenotype of *wnt1* ^*cre*^ *; Rosa26* ^*Dlx2/-*^ mice at E15.5 **(A)** Stereo microscope images showing the cleft (black arrow) in the palate at E15.5 **(B)** HE staining of frontal sections showing the morphology of maxillary processes of WT and *wnt1* ^*cre*^ *; Rosa26* ^*Dlx2/-*^ mouse embryos at E15.5. *Wnt1* ^*cre*^ *; Rosa26* ^*Dlx2/-*^ embryos display a cleft in the palate. White star indicates the towering tongue.

We next focused on understanding how Dlx2 overexpression in neural crest-derived mesenchymal cells affects the development of maxillary processes and causes short maxilla and cleft palate. We examined the morphology of maxillary processes of *wnt1* ^*cre*^ *; Rosa26* ^*Dlx2/-*^ mouse embryos at different developmental stages. At E15.5, all four *wnt1* ^*cre*^ *; Rosa26* ^*Dlx2/-*^ embryos from three litters (17 in total) across three generations displayed a depression-like cleft in the palate (Figure 4A, black arrow; Figure 4B), which penetrates the anterior and posterior of the palate. Notably, the tongue of E15.5 *wnt1* ^*cre*^ *; Rosa26* ^*Dlx2/-*^ mouse embryos also appeared towering (Figure 4B, white star). Such a phenotype may be due to the changes in the pressure of the palate, which caused the tongue to fill the space of the cleft.

**FIGURE 5:**
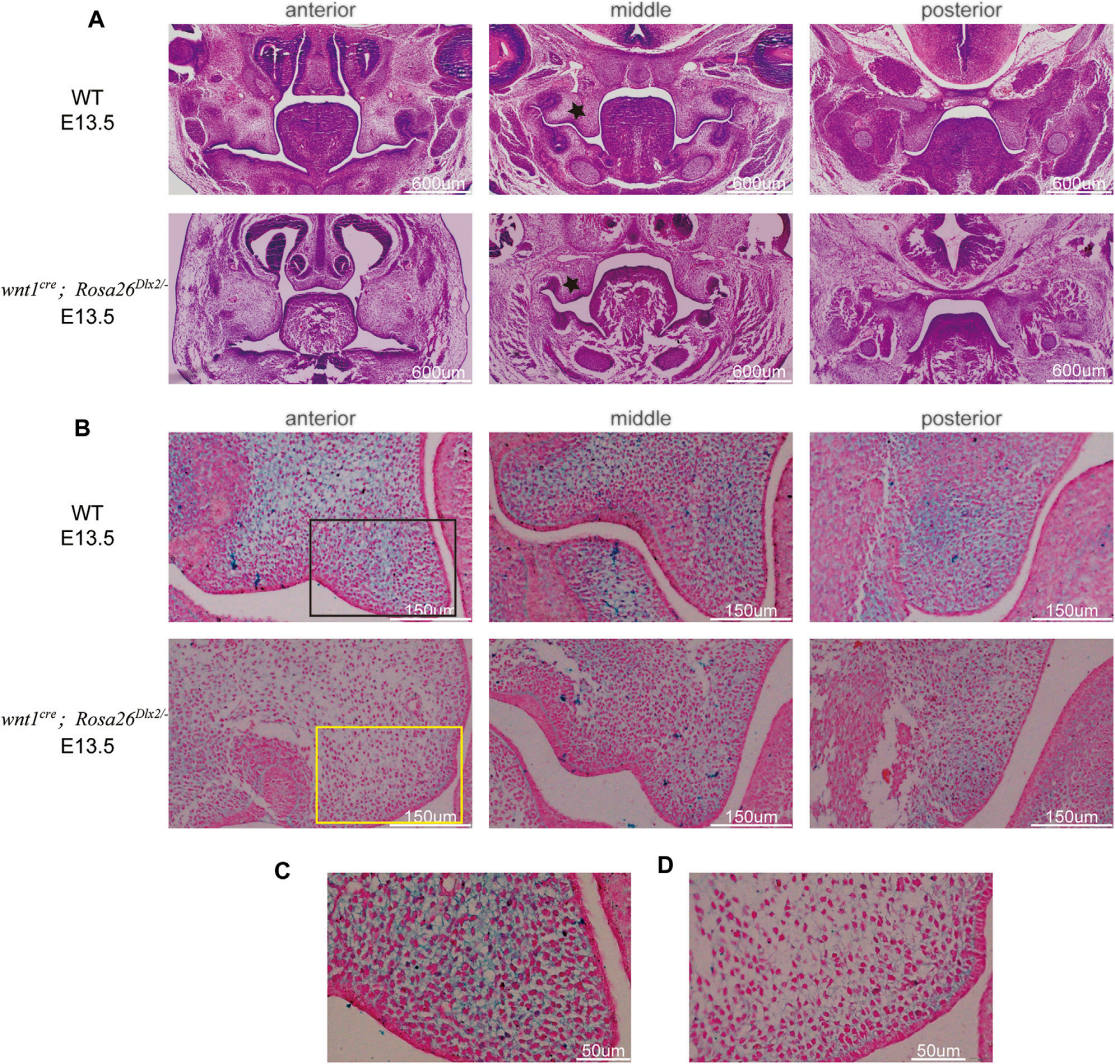
Conditional Dlx2 overexpression in *wnt1* ^*cre*^ *; Rosa26* ^*Dlx2/-*^ mice caused cleft palate by affecting maxillary growth and uplift **(A)** HE staining of frontal sections showing the morphology of maxillary processes of WT and *wnt1* ^*cre*^ *; Rosa26* ^*Dlx2/-*^ mouse embryos at E13.5. The maxillary processes of the *wnt1* ^*cre*^ *; Rosa26* ^*Dlx2/-*^ mice are smaller than those of WT. Black stars indicate the maxillary processes **(B)** HA staining of the right maxillary process at E13.5 showing HA expression in *wnt1* ^*cre*^ *; Rosa26* ^*Dlx2/-*^ mice is decreased compared with WT mouse embryos **(C,D)** Locally enlarged palatal process sections display that HA fails to fill the ECM space and cell density is decreased in *wnt1* ^*cre*^ *; Rosa26* ^*Dlx2/-*^ mouse embryos **(C)** Enlarged picture of the black box area in **B (D)** Enlarged picture of the yellow box area in **B**.

We next investigated whether the cleft palate in E15.5 *wnt1* ^*cre*^ *; Rosa26* ^*Dlx2/-*^ embryos resulted from defective palate fusion or maxillary growth. Because the palate fusion in mouse embryos occurs at E14.5, we compared the morphology of E13.5 *wnt1* ^*cre*^ *; Rosa26* ^*Dlx2/-*^ mice embryos with their WT littermates. At E13.5, the maxillary processes of the *wnt1* ^*cre*^ *; Rosa26* ^*Dlx2/-*^ mice were markedly smaller than those of the WT (Figure 5A, black star). These findings collectively suggested that the cleft palate phenotype in *wnt1* ^*cre*^ *; Rosa26* ^*Dlx2/-*^ mice cannot be solely attributed to defective palatal adhesion and fusion. Rather, the conditional Dlx2 overexpression already led to abnormal development of the maxillary process during the vertical outgrowth stage.

We used HA staining to analyze its accumulation. At E13.5, HA expression in *wnt1* ^*cre*^ *; Rosa26* ^*Dlx2/-*^ mice was decreased compared with WT mouse embryos (Figure 5B). Of note, in the anterior and middle regions of the palatal process of *wnt1* ^*cre*^ *; Rosa26* ^*Dlx2/-*^ mice, HA downregulation was more marked. The palatal process sections of WT and *wnt1* ^*cre*^ *; Rosa26* ^*Dlx2/-*^ mice stained with HA were locally enlarged to observe HA expression and cell density (Figure 5C,D). We found that WT mouse palatal mesenchymal extracellular matrix (ECM) was filled with HA. By constrast, in the palatal process of *wnt1* ^*cre*^ *; Rosa26* ^*Dlx2/-*^ mice, HA failed to fill the ECM space. In addition, an decrease in cell density was observed in the palatal process of *wnt1* ^*cre*^ *; Rosa26* ^*Dlx2/-*^ mice, indicating that the number of cells decreased and the growth of palatal process was damaged. Therefore, it is reasonable to propose that the mutation resulted in downregulation HA expression, which affects the filling of extracellular stroma of palatal process cells, leading to insufficient uplift of palatal process. In addition, the decrease in the number of mesenchymal cells leads to insufficient growth of palatal process tissue, and finally results in problems in the lifting and fusion of palatal process.

### Dlx2 Overexpression Causes Profound Gene Expression Changes During the Development of Maxillary Processes

We reasoned that Dlx2 overexpression may disrupt the complex gene expression programs during craniofacial development, thereby causing craniofacial defects. The *wnt1* ^*cre*^ *; Rosa26* ^*Dlx2/-*^ mice that exhibited consistent and synchronized cleft palate phenotype enable us to profile the impact of Dlx2 overexpression on the transcriptome of mouse maxillary processes.

**FIGURE 6:**
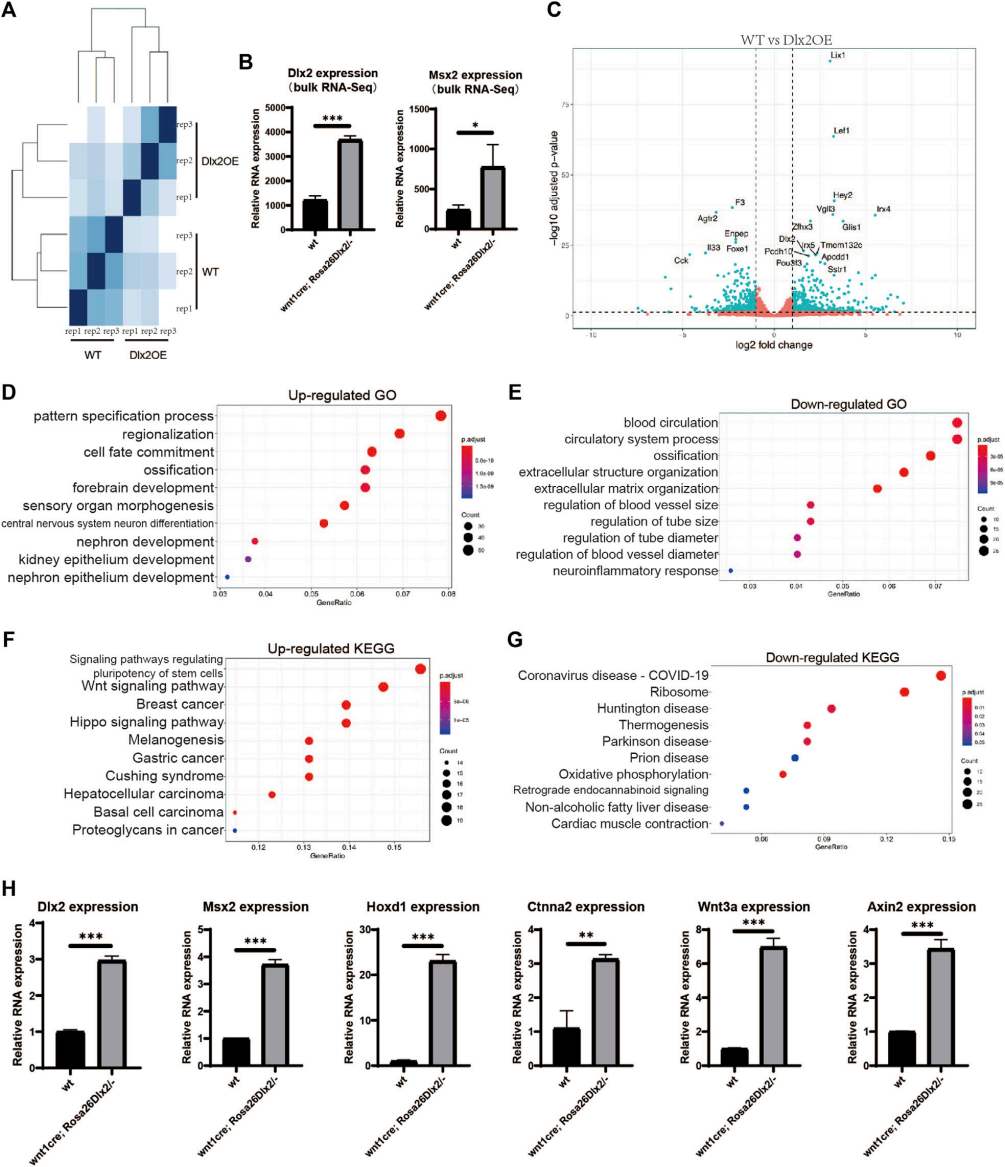
Bulk RNA-seq of the maxillary processes from E12.5 WT and *wnt1* ^*cre*^ *; Rosa26* ^*Dlx2/-*^ embryos **(A)** Sample distances matrix showing the correlation between RNA-seq replicates **(B)** Bar graphs showing the Dlx2 and Msx2 expression levels in WT and *wnt1* ^*cre*^ *; Rosa26* ^*Dlx2/-*^ samples according to bulk RNA-seq. **(C)** Volcano plots show differentially expressed genes between WT and *wnt1* ^*cre*^ *; Rosa26* ^*Dlx2/-*^ samples **(D,E)** GO enrichment analysis of genes significantly upregulated or downregulated in *wnt1* ^*cre*^ *; Rosa26* ^*Dlx2/-*^ **(F,G)** KEGG pathway enrichment results for genes significantly upregulated or downregulated in *wnt1* ^*cre*^ *; Rosa26* ^*Dlx2/-*^ **(H)** Bar graphs show the Msx2, Hoxd1, Ctnna2, Wnt3a and Axin2 expression levels in WT and *wnt1* ^*cre*^ *; Rosa26* ^*Dlx2/-*^ samples according to qPCR.

We surgically isolated maxillary processes from E12.5 WT or *wnt1* ^*cre*^ *; Rosa26* ^*Dlx2/-*^ embryos and performed bulk RNA-seq on three independent biological replicates for each genotype. The individual RNA-seq replicate exhibited a high degree of correlation with those of the same genotype, while correlating less well with the replicates of the different genotype, indicating the good quality of our RNA-seq datasets (Figure 6A). Analysis of the RNA-seq data revealed that Dlx2 was upregulated by approximately 3-fold in *wnt1* ^*cre*^ *; Rosa26* ^*Dlx2/-*^ than in WT. Moreover, Msx2, a transcription factor that is known to be a transcriptional target for Dlx2 (Sun, Liu, Li, Dai, & Wang, 2015; Dai et al., 2017; Zeng, Wang, Dong, Ma, & Liu, 2020), also exhibits a 3-fold upregulation upon Dlx2 overexpression (Figure 6B). The Msx2 expression in E12.5 maxillary process was confirmed by qPCR (Figure 6H), which was consistent with the bulk RNA-seq results. Collectively, these results demonstrate the efficacy of the Dlx2 overexpression in our novel mouse model and its value in elucidating the functions of Dlx2.

Comparison between the bulk results of *wnt1* ^*cre*^ *; Rosa26* ^*Dlx2/-*^ and WT mice revealed that 2428 genes exhibited significant expression changes, of which 447 genes were significantly upregulated and 440 genes were significantly downregulated (Figure 6C). The most upregulated genes in *wnt1* ^*cre*^ *; Rosa26* ^*Dlx2/-*^ mice include multiple transcription factors that have been implicated in craniofacial development, including Lef1, Irx4, and Pou3f3, demonstrating the critical functions of Dlx2 in orchestrating the transcriptional networks in neural crest-derived cells. Notably, Lef1, a transcription factor involved in regulating the Wnt pathway, has been shown to express at a high level in both epithelial and peri-epithelial mesenchyme during the early developmental stage of maxillary processes (Chen, Lan, Baek, Gao, & Jiang, 2009), consistent with the expression pattern of the WT Dlx2. Therefore, that a moderate Dlx2 upregulation is sufficient to induce marked remodeling of the transcriptional network and severe craniofacial defects, this highlights the critical functions of Dlx2 in regulating the development of craniofacial tissues.

The GO and KEGG enrichment analyses on the differentially regulated genes revealed that multiple development-related biological processes were impacted by Dlx2 overexpression. Genes associated with epithelial and neuronal development were enriched among the upregulated genes (Figure 6D), while genes associated with blood vessel development were enriched among the downregulated genes (Figure 6E). Hoxd1, an early neurodermal marker, which is part of a development regulatory system (Appukuttan et al., 2000), was significantly increased by qPCR(Figure 6H), which was consistent with the results of the bulk RNA-seq. Observing the down-regulated GO-term “exteacellular structure organization” and “extracellular matrix organization”, it can be found that their enriched genes contain Mmp9 (Dias et al., 2022), Ccn2 (Takeuchi-Igarashi, Tachibana, Murakashi, Kubota, & Numabe, 2021), Kazald1(H. Wang et al., 2013) and Col9a3(P. Zhang & Chang, 2020), which are genes related to cell proliferation. Therefore, the Dlx2 overexpression leads to the down-regulation of a group of genes related to cell proliferation, which hinders the development of palate by inhibiting cell proliferation. Notably, genes associated with the GO-term “ossification” were enriched among both the upregulated and downregulated genes upon Dlx2 overexpression, suggesting that the skeletal defects observed in *wnt1* ^*cre*^ *; Rosa26* ^*Dlx2/-*^ mice result from profound dysregulation of genes related to skeletal development.

KEGG enrichment analysis suggested that the genes involved in Wnt and Hippo signaling were enriched among the upregulated genes (Figure 6F), in agreement with the upregulation of the Lef1 gene described above, suggesting that Dlx2 may indeed play an important role in regulating the Wnt pathway during craniofacial development. Axin2 (Jho et al., 2002) and Wnt3a (Samarzija, Sini, Schlange, Macdonald, & Hynes, 2009), the classical reporter genes of the Wnt signaling pathway, were detected by qPCR. Their expressions were consistent with bulk RNA-seq, both being significantly increased (Figure 6H). Ctnna2, which is closely associated with Hippo signaling pathways (Pavel et al., 2021), also displayed a consistent upregulation (Figure 6H). Moreover, genes related to the KEGG terms “ribosome” and “oxidative phosphorylation” were significantly enriched among the downregulated genes (Figure 6G), implying that Dlx2 overexpression may cause decreases in cellular biosynthesis and energy metabolism, thereby inhibiting cellular growth during craniofacial development. In summary, our systematical characterization of the transcriptome changes induced by Dlx2 overexpression revealed the critical functions of Dlx2 in the regulation of a variety of development-related pathways, providing important information for understanding the regulatory functions of Dlx2 during craniofacial development.

## Discussion

In the present study, we report a novel Dlx2 conditional overexpression mouse model (*Rosa26* ^*CAG-LSL-Dlx2−3xFlag*^) that can be used to achieve stable, neural crest-specific Dlx2 overexpression by breeding with the wnt1^cre^ mice to obtain *wnt1* ^*cre*^ *; Rosa26* ^*Dlx2/-*^ mice. Compared to the previously established, high copy-number transgene-based Wnt1Cre:iZEG-Dlx2 mouse model, our mouse model confers several clear advantages. First, the extent of Dlx2 overexpression in *wnt1* ^*cre*^ *; Rosa26* ^*Dlx2/-*^ mice is only approximately 3-fold of the endogenous Dlx2 expression level, therefore more likely mimicking the pathological Dlx2 overexpression that may be caused by mutations in coding sequences of developmental genes or noncoding cis-regulatory elements. Second, the *wnt1* ^*cre*^ *; Rosa26* ^*Dlx2/-*^ mice exhibit highly consistent phenotypes that include cleft palate, maxillary shortness, and skull parietal defect among others. All these phenotypes of the *wnt1* ^*cre*^; *Rosa26* ^*Dlx2/-*^ mice are completely penetrative, drastically exceeding the less than 50% penetrance rate in Wnt1Cre:iZEG-Dlx2 mice. Third, the craniofacial abnormalities in Wnt1Cre:iZEG-Dlx2 mice manifested at a lower rate and the phenotype gradually weakened after maintaining the iZEG-Dlx2 founder mice over multiple generations, presumably due to the genetic instability or epigenetic silencing associated with the high copy-number transgenes. By contrast, the *Rosa26* ^*CAG-LSL-Dlx2−3xFlag*^ mice can be stably maintained without compromising the activity of the Dlx2-expression cassette. Consequently, the *wnt1* ^*cre*^ *; Rosa26* ^*Dlx2/-*^ exhibit stable phenotypes over many generations. In summary, the *Rosa26* ^*CAG-LSL-Dlx2−3xFlag*^ mice provide a more reliable and consistent model for investigating Dlx2 functions during development.

The phenotypes of *wnt1* ^*cre*^ *; Rosa26* ^*Dlx2/-*^ mice are similar to some extent to those previously reported in Wnt1Cre:iZEG-Dlx2 mice, thus confirming the critical regulatory functions in the tissues derived from the neural crest, particularly in the first branchial arch. However, it is also notable that the severity of craniomaxillofacial abnormalities resulting from the approximately 3-fold Dlx2 overexpression differs from that in Wnt1Cre:iZEG-Dlx2 mice. While different degrees of the split face and even split brain were observed in Wnt1Cre:iZEG-Dlx2 mice, the *wnt1* ^*cre*^ *; Rosa26* ^*Dlx2/-*^ mice exhibited only a slight fissure in the middle of the upper lip, but no obvious mid-face fissures. These observations indicate that the teratogenicity of Dlx2 is dose-dependent, and different craniomaxillofacial structures may exhibit differential sensitivity to the Dlx2 expression level.

Palatal process lifting is the least understood stage in the hard palate formation. The reasons for the failure of palatal process lifting are complex, and the HA has been intensively researched in recent years. HA accumulation is considered to be an important internal force in palatal process lifting (Yonemitsu, Lin, & Yu, 2020). It can bind a large number of water molecules, expand extracellular interstitial space, reduce cell density, increase tissue extension, and regulate the osmotic pressure of palatal process mesenchyme, and thus provides a driving force for palatal process lifting (Li, Lan, & Jiang, 2017). Insufficient HA accumulation is one of the important reasons for disorder in palatal process lifting. We observed that during the vertical growth and palatal process lifting of mutant mice, HA expression was significantly down regulated, and the extracellular stroma collapsed and did not obtain good filling. It was confirmed that Dlx2 can regulate HA accumulation during palatal process. In the palatal process during the growth and lifting stages, correct Dlx2 expression can promote HA expression and aggregation, thereby facilitating the extension and remodeling of palatal process mesenchymal tissue and providing an internal force for palatal process lifting. At the same time, many researchers have proved that HA is a downstream target of FGF family-related members, including FGFR1 and FGF2, in different organ development, disease occurrence and tissue regeneration (McCarthy, Sidik, Bertrand, & Eberhart, 2016; Virakul et al., 2016). The Wnt pathway can regulate the hyaluronic acid synthase family and mediate HA synthesis, thus participating in wound healing and embryonic mesodermal cell differentiation (Carre et al., 2010; Xu, Wang, Liu, Xie, & Chen, 2018). Therefore, Dlx2 is likely to affect the function of hyaluronic acid synthase and alter HA synthesis by regulating the FGF and Wnt pathways.

The consistent phenotypic manifestations of the *wnt1* ^*cre*^ *; Rosa26* ^*Dlx2/-*^ mice enable systematic elucidation of the effects of Dlx2 overexpression on transcriptomes at different stages of craniofacial development. Dlx2 has been confirmed to accelerate osteogenic differentiation both *in vitro* and *in vivo* (Y. Wang, Liu, Zhang, & Luo, 2020; Zeng et al., 2020; J. Zhang, Zhang, Dai, Wang, & Shen, 2019; J. Zhang, Zhang, Shi, Dai, & Shen, 2018). Previous studies have demonstrated that the spatiotemporally coordinated actions of Dlx and Msx homeobox transcription factors regulate skeletal growth and homeostasis (Lezot et al., 2010). By comparing the transcriptomes of maxillary processes from E12.5 WT and *wnt1* ^*cre*^ *; Rosa26* ^*Dlx2/-*^ mice, we showed that Msx2 transcription was upregulated upon Dlx2 overexpression. Furthermore, genes involved in ossification were significantly enriched among the upregulated genes, suggesting an intimate regulatory relationship between Dlx2 and Msx2 in the regulation of craniofacial skeletal development.

In addition to the regulation of osteogenesis, many genes related to neuronal development were significantly upregulated in E12.5 maxillary upon Dlx2 overexpression (Figure 6D). The functions of Dlx2 in neuron differentiation have been well documented (Al-Jaberi, Lindsay, Sarma, Bayatti, & Clowry, 2015; Alzu’bi & Clowry, 2019; Yang et al., 2017). While previous studies have demonstrated that Dlx2 promotes the differentiation of multiple types of interneurons, including olfactory bulb interneurons (Guo et al., 2019), basal forebrain (Le et al., 2017), and neocortical GABAergic interneurons (Al-Jaberi et al., 2015), the roles of Dlx2 in regulating peripheral nervous system development have not been fully investigated. Our results suggest that Dlx2 may act as an important regulator for craniofacial nerves during early development.

Our transcriptome analysis also associates Dlx2 with multiple critical developmental pathways. Dlx2 has been shown to regulate Wnt1 transcription (Yamada et al., 2016). A previous *in vitro* study in human bone mesenchyme stem cells demonstrated that Dlx2 promotes osteogenesis through the Wnt/β-catenin signaling pathway (Zeng et al., 2020). Consistent with this notion, we found that Wnt1 and the Wnt signaling transcription factor Lef1 are both upregulated upon Dlx2 overexpression. Therefore, the *wnt1* ^*cre*^ *; Rosa26* ^*Dlx2/-*^ mice provide a valuable model system for studying Wnt signaling regulation during craniofacial development. Moreover, we found that oxidative phosphorylation was repressed in the maxillary processes of *wnt1* ^*cre*^ *; Rosa26* ^*Dlx2/-*^ mice, in agreement with previous reports that the Dlx-2/Snail cascade is involved in glycolysis switch and mitochondrial repression in cancer cells (Lee et al., 2015) (Lee et al., 2019). Although this is not an example of Dlx2 in developmental science, Dlx2 inhibition of the oxidative phosphorylation process is consistent.

In conclusion, our findings highlight the complex transcriptional regulatory network downstream of Dlx2 and implicate Dlx2 in a wide array of developmental pathways. Our novel Dlx2 conditional overexpression mouse model and the transcriptome data thus offer valuable resources and insights for the future genetic elucidation of the transcriptional regulatory networks underlying craniofacial development.

## Data Availability

The data presented in the study are deposited in the National Center for Biotechnology Information (NCBI) Gene Expression Omnibus (GEO), accession number GSE185279.
